# Effects of individualized positive end-expiratory pressure combined with recruitment maneuver on intraoperative ventilation during abdominal surgery: a systematic review and network meta-analysis of randomized controlled trials

**DOI:** 10.1007/s00540-021-03012-9

**Published:** 2021-11-10

**Authors:** Xiang Li, Zhi-Lin Ni, Jun Wang, Xiu-Cheng Liu, Hui-Lian Guan, Ming-Sheng Dai, Xing Gao, Yang Zhou, Xiao-Yi Hu, Xun Sun, Jian Zhou, Qiu Zhao, Qian-Qian Zhang, He Liu, Yuan Han, Jun-Li Cao

**Affiliations:** 1 Jiangsu Province Key Laboratory of Anesthesiology, Xuzhou Medical University, Xuzhou, 221000 Jiangsu China; 2 Department of Thoracic Surgery, The Affiliated Hospital of Xuzhou Medical University, XuzhouJiangsu, 221000 China; 3 Department of Anesthesiology, Huzhou Central Hospital, Huzhou, 313000 Zhejiang China; 4 Department of Anesthesiology, Eye & ENT Hospital of Fudan University, Shanghai, 200031 China

**Keywords:** Individualized, Positive end-expiratory pressure, Alveolar recruitment maneuver, Protective lung strategy, Abdominal surgery, Network meta-analysis

## Abstract

Low tidal volume ventilation strategy may lead to atelectasis without proper positive end-expiratory pressure (PEEP) and recruitment maneuver (RM) settings. RM followed by individualized PEEP was a new method to optimize the intraoperative pulmonary function. We conducted a systematic review and network meta-analysis of randomized clinical trials to compare the effects of individualized PEEP + RM on intraoperative pulmonary function and hemodynamic with other PEEP and RM settings. The primary outcomes were intraoperative oxygenation index and dynamic compliance, while the secondary outcomes were intraoperative heart rate and mean arterial pressure. In total, we identified 15 clinical trials containing 36 randomized groups with 3634 participants. Ventilation strategies were divided into eight groups by four PEEP (L: low, M: moderate, H: high, and I: individualized) and two RM (yes or no) settings. The main results showed that IPEEP + RM group was superior to all other groups regarding to both oxygenation index and dynamic compliance. LPEEP group was inferior to LPEEP + RM, MPEEP, MPEEP + RM, and IPEEP + RM in terms of oxygenation index and LPEEP + RM, MPEEP, MPEEP + RM, HPEEP + RM, IPEEP, and IPEEP + RM in terms of dynamic compliance. All comparisons were similar for secondary outcomes. Our analysis suggested that individualized PEEP and RM may be the optimal low tidal volume ventilation strategy at present, while low PEEP without RM is not suggested.

## Introduction

Pulmonary gas exchange and respiratory mechanics are impaired during general anesthesia, due to the collapse of alveolar [1]. Conventional ventilation strategy with high tidal volume (*V*_T_) > 10 ml/kg predicted body weight, no positive end-expiratory pressure (PEEP), or routine recruitment maneuver (RM) was once recommended to maintain the alveolar open, but was recently proved ineffective and even related to severe volutrauma and barotrauma [2, 3]. The current view suggests that sufficient PEEP and routine RM based on low *V*_T_ (6-8 ml/kg predicted body weight) are the key points to maintain the alveolar open [2, 4, 5].

However, an excessive high PEEP may lead to barotrauma and hemodynamic instability; thus, the lowest PEEP that keeps the alveoli open is defined as “optimal PEEP”. The conclusions of previous studies showed a consistent controversy on the value of optimal PEEP [6–8]. At this background, a new concept named “individualized PEEP” was proposed, aiming at finding out the optimal PEEP according to patients’ individual characteristics such as lung dynamic compliance and driving pressure [9, 10].

The superiority of individualized PEEP over fixed PEEP was proved by recent studies in terms of intraoperative oxygenation index and respiratory mechanisms such as driving pressure and dynamic compliance [9–12], indicating less atelectrauma and barotrauma separately. However, the superiority was mostly concluded from the comparisons with moderate fixed PEEP (5–8 cm H_2_O) [13], while meta-analysis involving individualized PEEP is still absent. In addition, hemodynamic instability was another concern in the previous studies as individualized PEEP was usually higher than 10 cm H_2_O [9, 11].

We aimed at comprehensively evaluate the effects of individualized PEEP and RM based on low *V*_T_ ventilation strategy on intraoperative pulmonary function and hemodynamics during abdominal surgery, in comparisons of other PEEP and RM settings. Since PEEP (low, moderate, high, and individualized) and RM (yes or no) has multiple levels, the conventional pairwise comparison meta-analysis is difficult to achieve our purpose. We finally performed this network meta-analysis (NMA) and systematic review of randomized controlled trials (RCTs) to provide a strong evidence for the benefits of individualized PEEP.

## Materials and methods

The protocol for this NMA was registered with PROSPERO prospectively (identifier: CRD42020170614). The findings of this NMA was reported in accordance with the preferred reporting items for systematic reviews and meta-analyses–network meta-analyses (PRISMA-NMA) guidelines [14]. The PRISMA-NMA checklist can be found in Supplemental Digital Content 1.

### Search strategy

Two authors (QZ and QQZ) independently searched PubMed, EMBASE and Cochrane Library for eligible studies. The results were updated in December 23, 2020. Our keywords of PubMed were (((((((((((((("Tidal Volume"[Mesh]) OR Tidal Volumes) OR Volume, Tidal) OR Volumes, Tidal))) OR (((((((((((((((((("Positive-Pressure Respiration"[Mesh]) OR Positive-Pressure Respiration) OR Positive-Pressure Respirations) OR Respiration, Positive-Pressure) OR Respirations, Positive-Pressure) OR Positive-Pressure Ventilation) OR Positive-Pressure Ventilation) OR Positive-Pressure Ventilations) OR Ventilation, Positive-Pressure) OR Ventilations, Positive-Pressure) OR Positive End-Expiratory Pressure) OR End-Expiratory Pressure, Positive) OR End-Expiratory Pressures, Positive) OR Positive End-Expiratory Pressure) OR Positive End-Expiratory Pressures) OR Pressure, Positive End-Expiratory) OR Pressures, Positive End-Expiratory)))) OR recruitment maneuver)) AND Randomized Controlled Trial[Publication Type]) NOT (((animals [Mesh] not (humans [Mesh] and animals [Mesh])))))))) AND abdominal.

### Selection criteria

Two authors (ZLN and HLG) independently assessed the eligibility of studies by reading the titles, abstracts, and full texts. The chief investigator (JLC) arbitrated the disagreements and made final decisions. Studies were selected according to the following criteria:

(1) The participants were adult surgical patients undergoing supine position abdominal surgery requiring general anesthesia and low *V*_T_ ventilation on volume-control mode.

(2) The ventilation strategies were classified by levels of PEEP and RM.

(3) Included studies should report comparisons among two or more different low *V*_T_ ventilation strategies.

(4) We excluded studies that were not randomized controlled or written in English. Studies containing surgery in the lateral and prone position, and mechanical ventilation conducted by laryngeal mask also were excluded. However, we kept studies related to urology and gynecological surgery in the supine position.

### Data extraction and quality assessment

Two authors (XCL and JW) extracted the following data from the original full texts: first author, publication year, study design, procedure and type of surgery, patients’ characteristics [age, gender, body mass index (BMI), ASA class, and sample size], ventilation settings (*V*_T_, PEEP, and RMs), and intraoperative pulmonary function and hemodynamic indicators [oxygenation index, dynamic compliance, driving pressure, mean arterial pressure (MAP), and heart rate]. Oxygenation index is calculated by arterial partial pressure of oxygen/inspiratory oxygen fraction, and is determined by the ventilation and gas exchange function of patient's respiratory system. For patients with healthy lungs, the oxygenation index mainly depends on the degree of alveolar opening. Dynamic compliance is calculated by *V*_T_/(airway peak pressure – PEEP). Compared with the static compliance [*V*_T_/(airway plateau pressure—PEEP)] measured at the end of inspiration period, dynamic compliance represents not only elasticity of lung tissue, but also airway resistance which derives from periodic decruitment/recruitment alveoli and small airways [15, 16]. Thus dynamic compliance is associated with the degree of end-expiration alveolar opening. Driving pressure is calculated by (airway plateau pressure – PEEP), and reflects the degree of ventilator-induced barotrauma [17].

Continuous data were extracted as mean and standard deviation. The continuous data presented as median, interquartile range, and range was transferred to mean and standard deviation according to recommendations from the Cochrane Collaboration: assuming that the median was equivalent to the mean, the interquartile range and range was, respectively, divided by 1.35 and 4 to evaluate the standard deviation [18]. If data were merely available in graphical format, then GetData Graph Digitizer 2.25 (http://getdata-graph-digitizer.com/) was used to quantify it.

The Cochrane Collaboration tool containing randomization bias, allocation bias, subjects blinding bias, outcome blinding bias, incomplete data bias, and selective reporting bias was applied to assess the methodological quality independently by two authors.

### Outcomes

The primary outcomes were the intraoperative oxygenation index, dynamic compliance, and driving pressure. Secondary outcomes were the intraoperative heart rate and MAP.

### Statistical analysis

We performed this NMA by STATA13.1 (Stata Corporation, College Station, TX) to compare the effects of different PEEP and RM settings based on low V_T_ ventilation strategies on intraoperative oxygenation, lung dynamic compliance, heart rate, and MAP. The difference of mean, corresponding 95% CI, treatment rankings, probability of being best (Pr_Best_), and surface under the cumulative ranking curve (SUCRA) values were estimated using the random-effects model. For indirect comparisons, a node-splitting model was conducted to estimate the degree of inconsistency. *Z* test was performed to assess the significance of the overall effect size. *P* < 0.05 was considered statistically significant. To ensure the reliability of the NMA, the number of included RCTs was required to be at least equal to the number of ventilation strategies.

The frequency method was applied to the fitted meta-regression model after constructing a heterogeneity matrix. The model treats covariates as the basic parameters and presumes that heterogeneity is independent of the comparison between effect sizes from multi-arm studies. Inconsistency reflects the differences between direct and indirect effects for the same comparison. We estimated the probability of a treatment being ranked at a specific place using “network rank”. “Comparison-adjusted” funnel plot was used to evaluate the publication bias. The funnel plot should be symmetrical near the zero line if there is no publication bias. The certainty and hence quality of included studies in terms of within-study bias, reporting bias, indirectness, imprecision, heterogeneity, incoherence, and confidence rating was assessed according to the grading of recommendations assessment, development and evaluation (GRADE) system [19] using the CINeMA web app [20].

## Results

### Baseline characteristics of included studies

**Fig. 1:**
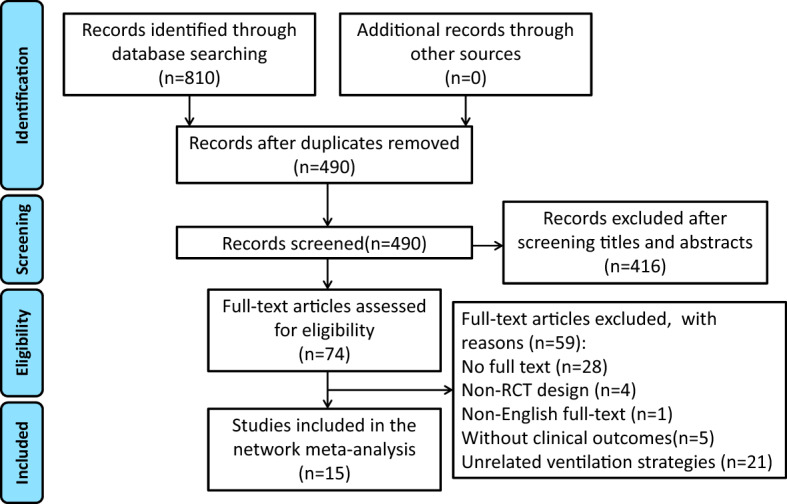
PRISMA flow diagram. *RCT* randomized controlled trial, *PRISMA* Preferred Reporting Items for Systematic Reviews and Meta-Analyses

The initial databases search identified 810 studies. Duplicates (*n* = 320), unrelated to mechanical ventilation (*n* = 296), protocol without data (*n* = 11), and intervention beyond our classification criteria (*n* = 109) were excluded after reviewing the title and abstract. Forty-six of the remaining 74 studies were available for full text. After full-text review, non-randomized design (*n* = 4), non-English full text (*n *= 1), without clinical outcomes (*n* = 5), and unrelated ventilation strategies (*n* = 21) were further eliminated. Fifteen RCTs (*n* = 3634) met our selection criteria [6, 8, 11–13, 21–30]. The screening and inclusion process is presented in Fig. 1. In particular: one study reported in data for open and laparoscopic surgery separately, so we divided into 2 separate studies in the NMA [11].

Among the 15 RCTs, patients were divided by levels of PEEP (low, < 5 cm H_2_O; moderate, 5–8 cm H_2_O; high, > 8 cm H_2_O; individualized, decided by titration trial) and RM (yes or no) into eight groups: low PEEP + RM (LPEEP + RM), low PEEP (LPEEP), moderate PEEP + RM (MPEEP + RM), moderate PEEP (MPEEP), high PEEP + RM (HPEEP + RM), high PEEP (HPEEP), individualized PEEP + RM (IPEEP + RM), and individualized PEEP (IPEEP). The detailed baseline characteristics of the included studies are summarized in Table 1.

**Table 1:** Main characteristics of the included studies in this network meta-analysis

Study	Type of surgery	Age (year)	Gender (male)	ASA class	BMI (kg/m^2^)	Sample size	*V*_T_ (ml/kg)	PEEP (cm H_2_O)	RM	RM pressure (cm H_2_O)	Strategy classification
You et al. [21]	Robot-assisted laparoscopic radical prostatectomy	65.7	25	N	N	25	8	5	No	No	MPEEP
		65.3	25			25	8	0	No	No	LPEEP
Van Hecke et al. [13]	Laparoscopic bariatric surgery	42	15	2–3	42	50	8	9.2 (Individualized)	No	No	IPEEP
		40	4		42	50	8	10	No	No	HPEEP
Bluth et al. [6]	Noncardiac, non-neurological surgery	48.6	295	1–3	44	989	7	12	YES	40–50	HPEEP + RM
		48.9	694		43.5	987	7	4	No	No	LPEEP
Wei et al. [22]	Laparoscopic radical gastrectomy	37	5	N	45	12	8	0	No	No	LPEEP
		35	5		48	11	8	0	YES	40	LPEEP + RM
		39	5		43	11	8	8	YES	40	MPEEP + RM
^a^Pereira et al. [11]	Laparoscopic surgery	N	N	1–2	N	10	6–7	13.2 (Individualized)	YES	40	IPEEP + RM
						10	6–7	4	YES	40	LPEEP + RM
^a^Pereira et al. [11]	Open abdominal surgery	N	N	1–2	N	10	6–7	10.1 (Individualized)	YES	40	IPEEP + RM
						10	6–7	4	YES	40	LPEEP + RM
Ciftci et al. [23]	Laparoscopic Cholecystectomy	47	10	N	26.9	30	8	0	No	No	LPEEP
		49.4	23		27.1	60	8	5–8	No	No	MPEEP
Sargin et al. [24]	Laparoscopic Cholecystectomy	39	8	1–2	26	35	8	0	No	No	LPEEP
		43	10		27	35	8	5	No	No	MPEEP
		38	5		27	35	8	10	No	No	HPEEP
Nestler et al. [12]	Laparoscopic surgery	44.9	8	1–3	48.3	25	8	18.5 (Individualized)	YES	50	IPEEP + RM
		46.2	8		53.8	25	8	5	No	No	MPEEP
Ferrando et al. [25]	Open abdominal surgery	61	9	N	24	18	6	8 (Individualized)	YES	40	IPEEP + RM
		11	8		26	18	6	5	YES	40	MPEEP + RM
Chin et al. [26]	Robot-assisted laparoscopic radical prostatectomy	65.1	19	N	25.7	19	8	0	No	No	LPEEP
		63	19		25.5	19	8	8	No	No	MPEEP
Ahn et al. [27]	Robot-assisted laparoscopic radical prostatectomy	62	30	N	N	30	6	15	No	No	HPEEP
		63	30			30	6	15	YES	40	HPEEP + RM
Pi et al. [28]	Open abdominal surgery	69.7	13		22.3	21	7	8	YES	30	MPEEP + RM
		66.1	10		22.1	20	7	8	NO	NO	MPEEP
Schultz et al. [31]	Open abdominal surgery	65	259	1–5	25.5	445	8	12	YES	No	HPEEP + RM
		66	255		25.6	449	8	2	No	No	LPEEP
Russo et al. [29]	Laparoscopic gynecologic surgery	32.2	0	1	22.6	20	8	0	No	No	LPEEP
		34	0		22.1	20	8	5	No	No	MPEEP
		33.4	0		22.3	20	8	10	No	No	HPEEP
Kwak et al. [30]	Laparoscopic Cholecystectomy	41.8	13	N	N	30	8	0	No	No	LPEEP
		43.3	12			30	8	10	No	No	HPEEP

*ASA* American Society of Anesthesiologists, *BMI* body mass index, *N* not reported, *PnP* pneumoperitoneum, *LPEEP* low PEEP, *MPEEP* moderate PEEP, *HPEEP* high PEEP, *IPEEP* individualized PEEP; RM, recruitment maneuver; V_T_, tidal volume

^a^This study reported data of open and laparoscopic surgery separately, so we divided this study into 2 separate ones

In this NMA, three studies were included in the IPEEP + RM group. All of them titrated the individualized PEEP through the RM-decremental titration trial-RM process, which consisted of one RM before and after the decremental PEEP titration trial separately [11, 12, 25]. The detailed steps are as follows: first, patients received an RM, usually with an open-lung pressure of 40 cm H_2_O and a higher one in obese patients, to open the alveoli. Then, a decremental PEEP titration trial was performed by decreasing PEEP step by step from a high level (20 or 25 cm H_2_O) until 5 cm H_2_O. The individualized PEEP was defined as the PEEP with optimal value of the titration parameter (electrical impedance tomography-related parameters in two studies [11, 12] and dynamic compliance in one study [25]). For prevention of the alveoli re-decruitment at the end of titration, another RM was performed before the application of individualized PEEP.

One study was included in the IPEEP group [13]. Its titration process was an incremental PEEP titration trial without RM, which had a same definition of individualized PEEP as the decremental trial.

### Quality assessment

**Fig. 2:**
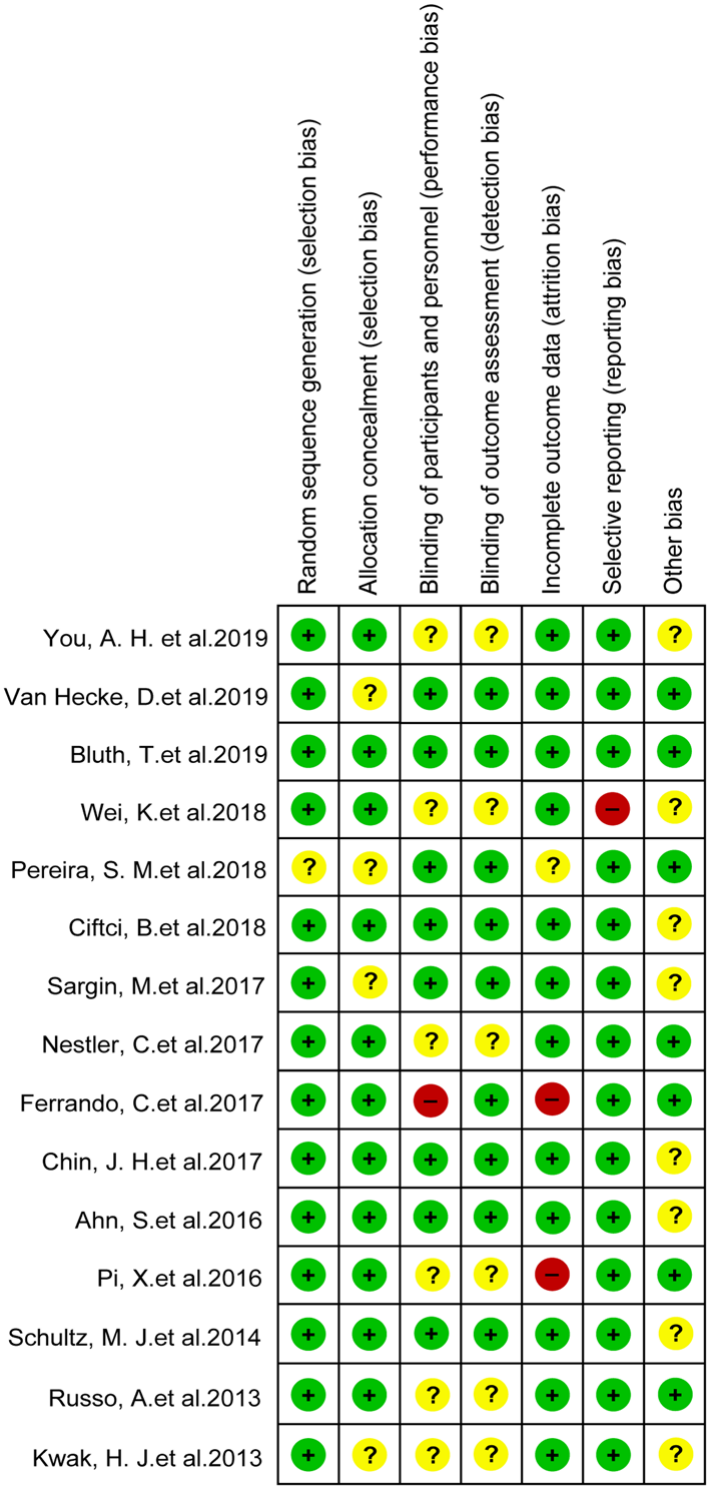
Risk-of-bias assessment of included studies

The details of the risk-of-bias assessment are summarized in Fig. 2. Fourteen studies reported clear randomization. Eleven studies reported allocation measures. Blinding methods for participants were absent in 7 studies and 6 studies lacked blinding methods for outcome assessors. Incomplete data were identified in 3 researches and selective reporting bias existed in 1 study.

The quality of evidence assessed by the GRADE system was moderate in all primary and secondary outcomes.

### Evidence network

**Fig. 3:**
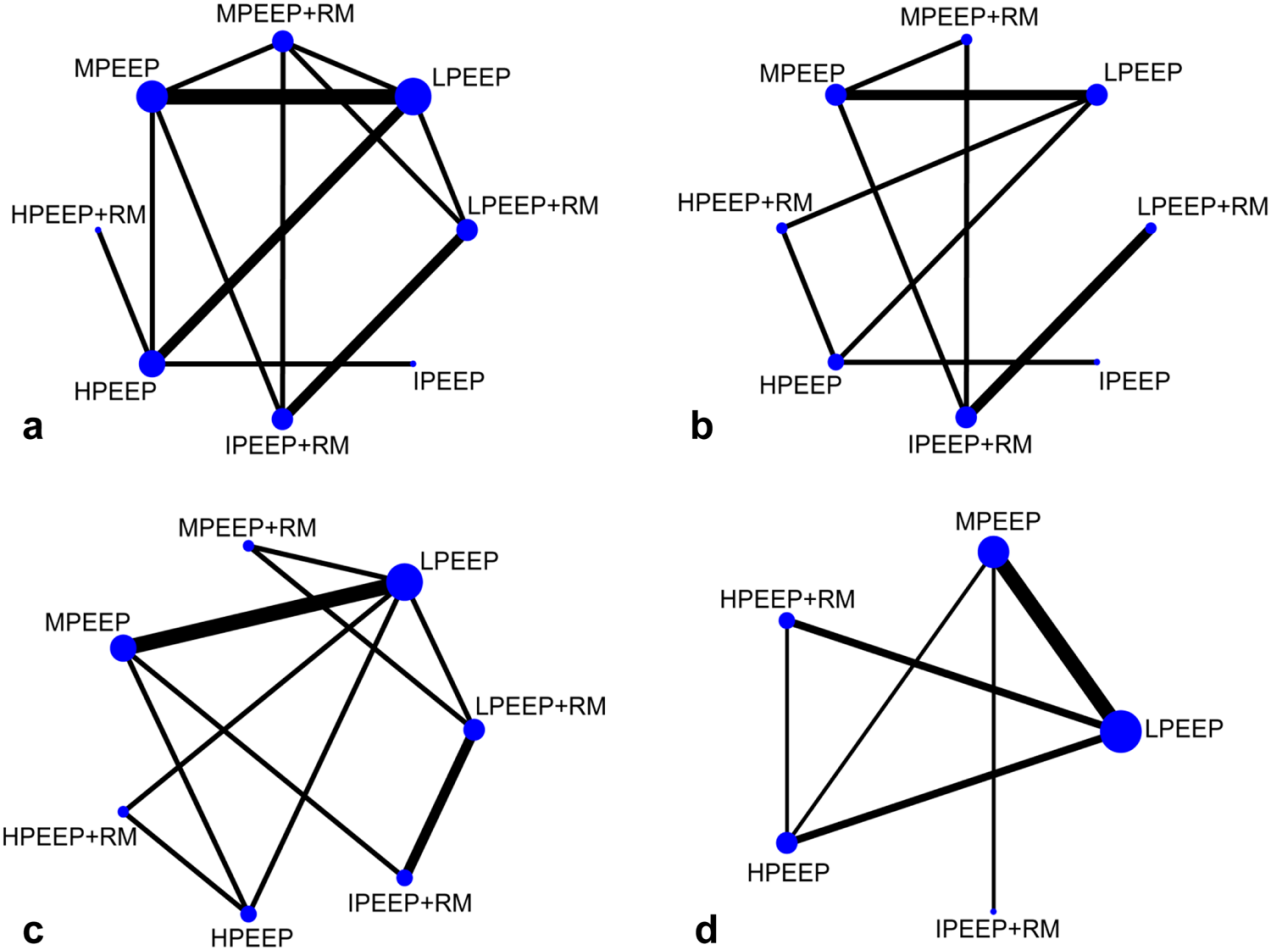
Network plot of enrolled studies in this network meta-analysis. **a** Oxygenation index; **b** dynamic compliance; **c** mean arterial pressure; **d** heart rate; *LPEEP* low PEEP, *MPEEP* moderate PEEP, *HPEEP* high PEEP, *IPEEP* individualized PEEP, *RM* recruitment maneuver

All network plots are presented in Fig. 3. Connecting lines indicated direct comparison, and indirect comparison among interventions can be performed by NMA. The size of nodes represents the overall sample size of each ventilation strategy and the width of connecting lines reflects the number of trials.

The network plots consisted of 5 triangular loops for oxygenation index, 2 triangular loops for dynamic compliance, 3 triangular loops for MAP, and 2 triangular loops for heart rate. For indirect comparisons, a node-splitting model was performed to estimate the degree of inconsistency. Inconsistency was found statistically significant for oxygenation index (*P* < 0.001 for loop moderate PEEP–moderate PEEP + RM–individualized PEEP + RM) and MAP (*P* = 0.016 for loop low PEEP–moderate PEEP–high PEEP, *P* = 0.035 for loop low PEEP–high PEEP + RM–high PEEP).

### Primary outcomes

#### Oxygenation index

12 RCTs of 569 patients were included and all 8 groups were available for the intraoperative oxygenation index. The IPEEP + RM group was found out superior to all other groups (Table 2). The LPEEP group was proved to be inferior to the LPEEP + RM, MPEEP, and MPEEP + RM group. Furthermore, the IPEEP group was shown to be inferior to the MPEEP + RM group. The SUCRA, Pr_Best_, and mean rank were: 100, 99.9%, and 1.0 for IPEEP + RM; 14.5, 0.0%, and 7.0 for LPEEP; 8.8, 0.0%, and 7.4 for IPEEP (Table 3).

**Table 2:**
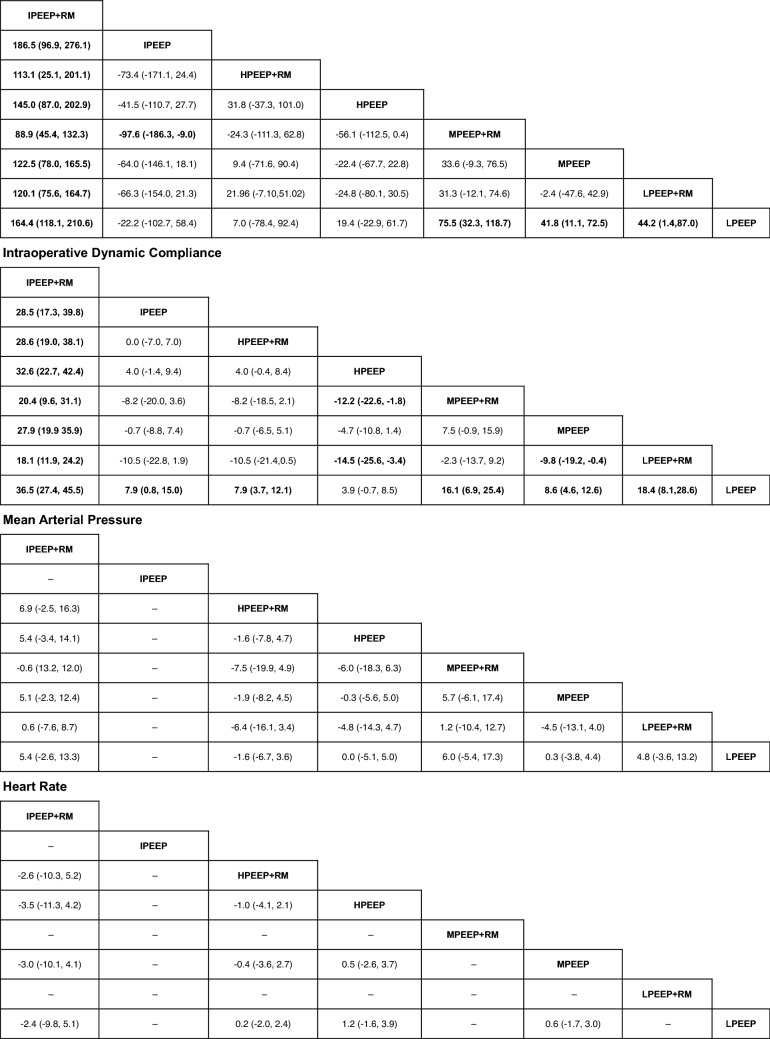
Network league table for all ventilation strategies in regard to intraoperative oxygenation index, dynamic compliance, mean arterial pressure, and heart rate

Estimates are presented as mean differences (95% confidence interval). Mean differences < 0 favor the column ventilation strategy and mean differences > 0 favor the row ventilation strategy

*LPEEP* low PEEP, *MPEEP* moderate PEEP, *HPEEP* high PEEP, *IPEEP* individualized PEEP, *RM* recruitment maneuver

Mean differences in bold are significantly different

**Table 3:** SUCRA, Pr_Best_, and mean rank of all ventilation strategies

Treatment	Oxygenation index			Dynamic compliance			Mean arterial pressure			Heart rate		
	SUCRA	Pr_Best_	MeanRank	SUCRA	Pr_Best_	MeanRank	SUCRA	Pr_Best_	MeanRank	SUCRA	Pr_Best_	MeanRank
LPEEP + RM	54.4	0.0	4.2	79.1	0.0	2.5	72.5	21.2	2.7	–	–	–
LPEEP	14.5	0.0	7.0	0.8	0.0	7.9	34.4	0.6	4.9	42.1	3.4	3.3
MPEEP + RM	78.9	0.0	2.5	73.5	0.0	2.9	76.1	46.4	2.4	–	–	–
MPEEP	52.6	0.0	4.3	45.9	0.0	4.8	36.1	0.2	4.8	61.6	22.6	2.5
HPEEP + RM	58.0	0.1	3.9	42.3	0.0	5.0	20.5	0.7	5.8	48.3	12.9	3.1
HPEEP	32.9	0.0	5.7	16.2	0.0	6.9	34.7	2.3	4.9	74.8	46.5	2.0
IPEEP + RM	100	99.9	1.0	100	100	1.0	75.6	28.6	2.5	23.1	14.6	4.1
IPEEP	8.8	0.0	7.4	42.1	0.0	5.1	–	–	–	–	–	–

*LPEEP* low PEEP, *MPEEP* moderate PEEP, *HPEEP* high PEEP, *IPEEP* individualized PEEP, *RM* recruitment maneuver, *Pr*_*Best*_ probability of being best, *SUCRA* surface under cumulating ranking curve

#### Dynamic compliance

11 RCTs of 1369 patients were included and all 8 groups were available for the dynamic compliance. The IPEEP + RM was found out superior to all other groups (Table 2). The LPEEP group was proved to be inferior to all other groups except the HPEEP group. Furthermore, the HPEEP group was shown to be inferior to the MPEEP + RM and LPEEP + RM group, while the MPEEP group was inferior to the LPEEP + RM group. The SUCRA, Pr_Best_, and mean rank were: 100, 100%, and 1.0 for IPEEP + RM; 0.8, 0.0%, and 7.9 for LPEEP (Table 3).

#### Driving pressure

There were only 4 studies that reported driving pressure, so the data were insufficient for NMA. The conclusion of raw studies suggested that driving pressure in IPEEP + RM group was lower than that in LPEEP + RM (*P* < 0.001), moderate PEEP (*P* < 0.001), and moderate PEEP + RM (*P* < 0.001) [6, 11, 12, 22]. The ventilation strategies, driving pressure, and P values are presented in Table 4.

**Table 4:** Driving pressure of included studies

Study	Type of surgery	Strategy classification	Driving pressure	*P*
^a^Pereira et al. [11]	Laparoscopic surgery	IPEEP + RM	7.1(0.9)	< 0.001
		LPEEP + RM	11.8(1.8)	
^a^Pereira et al. [11]	Open abdominal surgery	IPEEP + RM	7.8(1.4)	0.105
		LPEEP + RM	9.2(2.1)	
Nestler et al. [12]	Laparoscopic surgery	IPEEP + RM	7.1(1.4)	< 0.001
		MPEEP	13.7(2.6)	
Ferrando et al. [25]	Open abdominal surgery	IPEEP + RM	5.6(1)	< 0.001
		MPEEP + RM	7.4(1)	
Bluth et al. [6]	Open abdominal or laparoscopic surgery	HPEEP + RM	11.8(4.6)	< 0.001
		LPEEP	17.4(5.4)	

*LPEEP* low PEEP, *MPEEP* moderate PEEP, *HPEEP* high PEEP, *IPEEP* individualized PEEP, *RM* recruitment maneuver

^a^This study reported data of open and laparoscopic surgery separately, so we divided this study into 2 separate ones

### Secondary outcomes

#### Mean arterial pressure

In regard to the mean arterial pressure, 10 RCTs of 2413 patients were included and all groups except IPEEP were available. The mean differences of all direct and indirect comparisons showed no significant difference (Table 2). The SUCRA, Pr_Best_, and mean rank of all comparisons are presented in Table 3.

#### Heart rate

In regard to the heart rate, 9 RCTs of 3323 patients were included. Only LPEEP, MPEEP, HPEEP, HPEEP + RM, and IPEEP + RM groups were available. The mean differences of all direct and indirect comparisons showed no significant difference (Table 2). The SUCRA, Pr_Best_, and mean rank of all comparisons are presented in Table 3.

### Effects of recruitment maneuver

The mean differences between groups with same PEEP but different RM settings were merely significant in IPEEP + RM vs. IPEEP and LPEEP + RM vs. LPEEP, regarding to both intraoperative oxygenation index and dynamic compliance (Table 2).

The SUCRA, Pr_Best_, and mean rank of both two primary outcomes were superior in all groups with RM than that in groups with same PEEP but without RM (Table 3).

### Publication bias

**Fig. 4:**
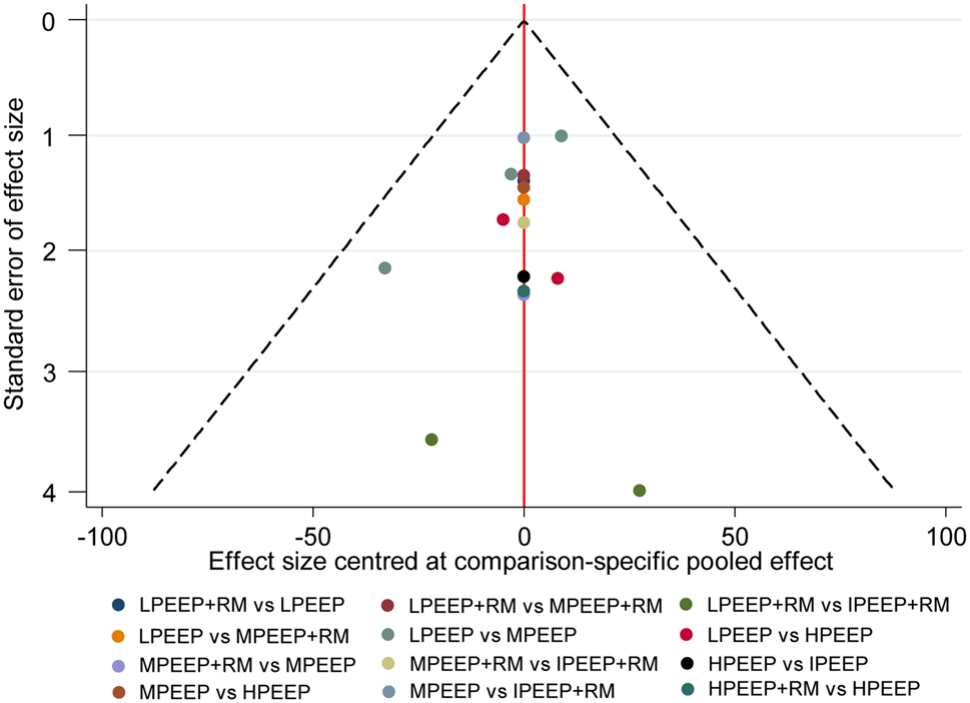
Comparison-adjusted funnel plot for intraoperative oxygenation index. *LPEEP* low PEEP, *MPEEP* moderate PEEP, *HPEEP* high PEEP, *IPEEP* individualized PEEP, *RM* recruitment maneuver

**Fig. 5:**
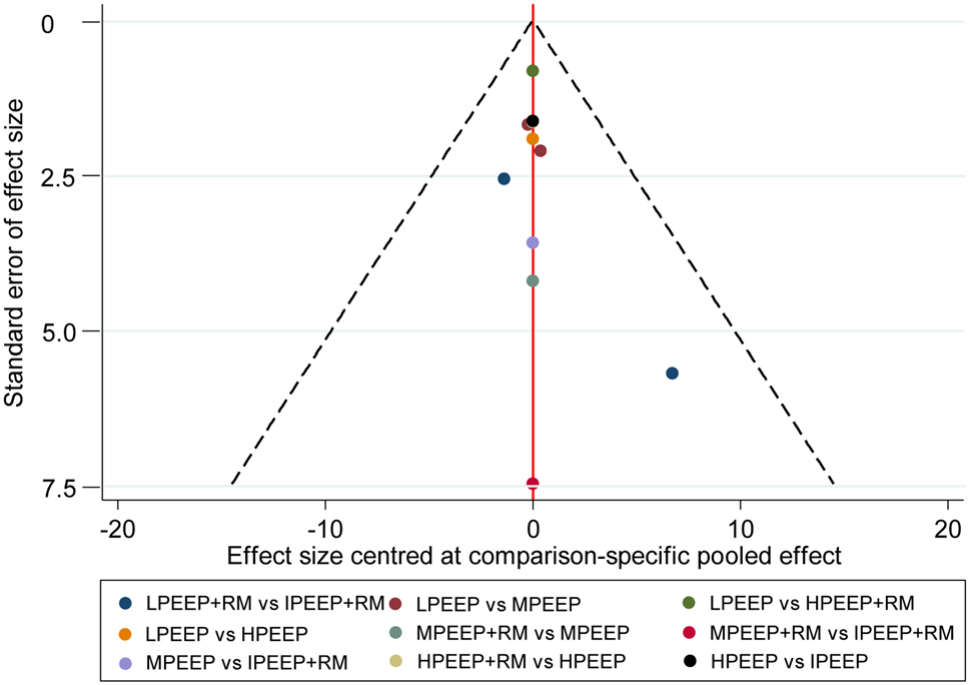
Comparison-adjusted funnel plot for intraoperative dynamic compliance. *LPEEP* low PEEP, *MPEEP* moderate PEEP, *HPEEP* high PEEP, *IPEEP* individualized PEEP, *RM* recruitment maneuver

The funnel plot of both primary outcomes is presented in Fig. 4 (oxygenation index) and Fig. 5 (dynamic compliance). The included studies were symmetrically distributed on both sides of the vertical line (*x* = 0), indicating no significant publication bias.

## Discussion

The present NMA demonstrate that in terms of intraoperative pulmonary function and hemodynamics, individualized PEEP combined with RM may be the currently optimal low *V*_T_ ventilation strategy, while low PEEP without RM was the worst low *V*_T_ ventilation strategy. Also, evidence suggested that RM was associated with improvements in oxygenation index and dynamic compliance.

The lung-protective ventilation strategy (*V*_T_ of 6 ml/kg, appropriate PEEP and RM) was proved superior to the conventional high *V*_T_ ventilation strategy on the prognosis of acute respiratory distress syndrome (ARDS) patients, as a consequence of reduced volutrauma and barotrauma [3]. Inspired by this result, researchers tried to apply lung-protective ventilation strategy in patients undergoing intraoperative mechanical ventilation. However, there are two main problems exposed in clinical anesthesia. First, a low *V*_T_ of 6 ml/kg was reasonable for ARDS patients to avoid excessive airway pressure but unnecessary for patients with healthy lungs. In addition, a tidal volume of 6 ml/kg is too low to be accepted by most anesthesiologists, compared with the traditional tidal volume of 10 ml/kg or higher. Therefore, most studies related to intraoperative ventilation strategy selected 8 ml/kg in groups receiving low tidal volume ventilation. Second, the low *V*_T_ ventilation strategy also increased the risk of atelectasis due to insufficient PEEP. In the previous studies, low V_T_ combined with low PEEP was associated with more serious atelectrauma and higher 30-day mortality in patients undergoing abdominal surgery and indicated the necessity of “optimal PEEP” setting [31]. Despite a series of efforts, no agreement has been reached on the optimal level of PEEP [6, 8, 31]. This debate may be attributed to applying one fixed PEEP to all patients, as their individual characteristics required different optimal PEEP. The concept of ‘individualized PEEP’ was then proposed to explore a more reasonable ventilation strategy [11].

Our NMA indicated a consistent strength of applying RM followed by individualized PEEP over other fixed PEEP strategies. The recent RCTs mainly revealed the advantages of individualized PEEP over low PEEP and moderate PEEP [9, 11, 32], while our NMA extended the conclusion to other fixed PEEP values. Higher oxygenation index and dynamic compliance suggested fewer collapsed alveoli. Based on current studies, two crucial points of the individualized PEEP may contribute to the alveoli opening effect. One is the variability of individualized PEEP. Even in non-obese patients undergoing open abdominal surgery, individualized PEEP could reach over 12 cm H_2_O. Such a high PEEP should be able to maintain most alveoli open. However, one included study revealed that compared with fixed PEEP of 2 cm H_2_O, a fixed PEEP of 12 cm H_2_O combined with RM did not lead to better intraoperative pulmonary function [6]. This contradictory conclusion can be explained by the other feature, large range of the individualized PEEP which is mostly over 10 cm H_2_O. The variability of individualized PEEP was probably caused by the individual characteristics such as chest wall compliance, abdominal pressure, pleural pressure, and surgical position. These inevitable high inter-individual heterogeneities strengthen the importance of individualizing the optimal PEEP.

The other crucial point of individualized PEEP is the correct titration process. In this NMA, all three studies in the IPEEP + RM group selected the RM-decremental titration trial-RM process [11, 12, 25]. The individualized PEEP was defined as the PEEP with optimal value of the titration parameter (electrical impedance tomography-related parameters in two studies [11, 12] and dynamic compliance in one study [25]). The included study in the IPEEP group used the incremental PEEP titration without RM, with the same definition of individualized PEEP as the decremental trial [13].

In our current study, the IPEEP + RM group was superior to IPEEP group in terms of both oxygenation index and dynamic compliance. One previous mathematical model revealed the possible reason that there was a consistent relationship between the PEEP level giving maximum lung compliance and the preset “open lung PEEP” (i.e., optimal PEEP) in the decremental trial with RM. This relationship, however, was inconsistent in the incremental trial without RM [33]. This hypothesis was later confirmed by a clinical trial related to obese patients with ARDS [34]. Therefore, we concluded that the individualized PEEP should be titrated under the RM-decremental titration-RM trial for consideration of higher intraoperative oxygenation and lung compliance.

Our NMA showed that RM improved the intraoperative oxygenation index and dynamic compliance in groups with RM especially in individualized PEEP and low PEEP groups, which were in line with previous RCTs [35, 36]. RM improved pulmonary function by overcoming the opening pressure, reverse atelectasis, and promote the benefits of PEEP [37]. The PEEP value applied during mechanical ventilation was usually insufficient for alveolar recruitment [38]; thus, a large amount of dorsal alveoli near the diaphragm may maintain collapsed without RM. In addition, evidence showed that even if RM was absent, the end-expiratory lung volume still increased along with the elevated level of PEEP [34]. That mean applying PEEP without RM ventilated air into the already open alveoli, inducing volutrauma and barotraumas and the injury was more severe when using higher PEEP. As the individualized PEEP is usually at a high level, the importance of RM should be considered seriously.

Based on the aforementioned effects of PEEP and RM, it is reasonable to believe that the low PEEP group has the worst oxygenation index. In the case of low V_T_, insufficient PEEP and lack of RM significantly increase the amount of atelectasis, leading to the impairment of gas exchange and respiratory mechanism. Previous studies also revealed that simply lowering V_T_ without supplementing PEEP and RM would significantly increase the incidence of PPCs and even mortality in surgical patients, in comparison of high V_T_, zero PEEP, and no RM strategy [31, 39].

Our analysis of MAP and heart rate revealed a limited impact of different PEEP and RM settings on intraoperative hemodynamic, which is similar to previous studies. It is worth noting that hypotension is common during the titration trial of individualized PEEP though proved one-past and harmless [11, 12]. The possible reason for hemodynamic instability when applying individualized PEEP is that the appropriate level of PEEP keeps most alveoli open, improves oxygenation, and thus reduces pulmonary vascular resistance and right ventricular load [40].

This study is a relatively small NMA. Its limitations are as follows: first, we only included studies related to low V_T_ ventilation strategies, without studies about conventional high V_T_ strategy. Second, the analysis of the incidence of PPCs was absent, which should be an issue worth more attention than oxygenation and lung compliance. Of note, individualized PEEP was previously proved to increase mortality in patients with moderate-to-severe ARDS [41]. Furthermore, the maneuver of RM–titration–RM may induce airway peak pressure to reach even 40 cm H_2_O. It would inevitably lead to alveolar barotrauma, which is fatal for patients with severe lung injury. The data we initially extracted included the incidence of PPCs, but only 5 included studies reported the events of PPCs, and the data were insufficient for a proper NMA or even a conventional pairwise comparison meta-analysis. Therefore, further evidence is necessary before applying individualized PEEP as a routine strategy, and future studies should focus on the incidence of PPCs. Thirdly, two studies have much larger sample sizes than other studies [6, 8], which may increase the risk of type I and II errors. Fortunately, both two studies lack oxygenation index data, thus not included in the analyses of primary outcome.

## Conclusion

In conclusion, our systematic review and NMA suggested that individualized PEEP combined with recruitment maneuver may be the optimal low *V*_T_ ventilation strategy in abdominal surgery at present, while low PEEP without recruitment maneuver may be the worst one. Recruitment maneuver is able to improve the intraoperative pulmonary function in all PEEP levels. In our opinion, further research should focus on the direct comparison of the individualized PEEP to the high PEEP, in terms of not only functional but also “hard-core” indicators such as the incidence of PPCs and mortality.

## Data Availability

The datasets generated and/or analyzed during the current study are available from the corresponding author on reasonable request.

## Abbreviations

ARDS: Acute respiratory distress syndrome
MAP: Mean arterial pressure
NMA: Network meta-analysis
PEEP: Positive end-expiratory pressure
PPC: Postoperative pulmonary complication
Pr_Best_: Probability of being best
RCT: Randomized controlled trial
RM: Recruitment maneuver
SUCRA: Surface under cumulative ranking curve
*V*_T_: Tidal volume
